# What’s new in the management of adult bronchiectasis?

**DOI:** 10.12688/f1000research.10613.1

**Published:** 2017-04-20

**Authors:** Usma Koser, Adam Hill

**Affiliations:** 1 Department of Respiratory Medicine, Royal Infirmary and University of Edinburgh, 51 Little France Crescent, Old Dalkeith Road, Edinburgh, EH16 4SA, UK

**Keywords:** Bronchiectasis, NCFB, Non-Cystic Fibrosis bronchiectasis

## Abstract

Bronchiectasis is a heterogeneous, chronic condition with many aetiologies. It poses a significant burden on patients and healthcare practitioners and services. Clinical exacerbations often result in reduced quality of life, increased rate of lung function decline, increased hospitalisation, and mortality. Recent focus in respiratory research, guidelines, and future management options has improved this clinical field in evidence-based practice, but further work and phase III clinical trials are required. This article aims to summarise and explore advances in management strategies in recent years and highlight areas of research and future focus.

## Introduction

Non-cystic fibrosis bronchiectasis (NCFB) is a chronic inflammatory condition resulting from repeated insult and/or obstruction to small and medium-sized bronchi, leading to fixed dilation and architectural distortion ^1, 2^. The clinical presentation varies from a chronic, productive, daily cough and recurrent infections to haemoptysis, dyspnoea, and respiratory failure ^2, 3^.

Bronchiectasis has diverse aetiologies, including idiopathic (up to 50% of cases), post-respiratory tract infection, rare immunodeficiency disorders, genetic abnormalities, autoimmune conditions, chronic inflammation, and mechanical obstruction ^2, 3^. It is also increasingly being recognised as part of a disease entity coinciding with and complicating other pulmonary conditions such as chronic obstructive pulmonary disease (COPD).

Prevalence rates fluctuate globally, between age groups, and between the sexes and are still largely unknown. However, a recent publication suggests that there is increasing overall incidence and prevalence ^4^. In the UK, overall incidence increased from 21 to 35 in women and 18 to 26 in men per 100,000 patient years between 2004 and 2013. The prevalence rates in women increased from 350 to 566 per 100,000 patient years and in men increased from 301 to 485 per 100,000 patient years in the same time ^4^. This may be, in part, due to increased diagnosis of the condition and improved computed tomography techniques. The study also illustrated the increased mortality rates in this population group and the need for hospitalisation, further strengthening the case for the increasing impact it has on our healthcare systems ^4^.

The mainstay of management is improving symptoms and reducing exacerbations. This article will cover idiopathic and post-infective bronchiectasis in adults and will not include allergic bronchopulmonary aspergillosis (ABPA), cystic fibrosis (CF), immunodeficiency, and non-tuberculous mycobacterium (NTM) disease. It will focus on the therapeutic strategies in bronchiectasis management, particularly highlighting new evidence from between 2013 and 2016.

## The patient-led approach

The first approach to management is to treat the underlying cause if one is identifiable ^3, 5, 6^. British Thoracic Society (BTS) guidelines have a systematic method for screening patients for ABPA, immunodeficiency, CF, and environmental tuberculosis (TB) infection ^6^.

The importance of risk stratification has been highlighted in recent years ^7, 8^. Two main scoring systems have been developed to illustrate severity and mortality – the bronchiectasis severity index (BSI) and FACED ^9, 10^. Both include FEV _1_ (forced expiratory volume in 1 second), dyspnoea, *Pseudomonas* colonisation, and radiological features. They have their limitations, mainly sample size and disparity between mild and moderate phenotypes, but are deemed suitable to predict mortality ^9, 10^. Additionally, the BSI also includes predictors for hospitalisation and annual mortality risk ^9^. These may be useful as adjuncts to decide on clinical management, but further studies are needed to see if such screening systems can be utilised in this way.

The vicious cycle hypothesis was first introduced by Cole and moulds our management interjections ^11^. Failure of mucus clearance, persistent infection, and inflammation leading to structural damage are all key aspects. Breaking this cycle by optimising interventions via airway clearance and antibiotic therapy for acute infections has been a backbone of treatment ^11^. Broadly categorising therapies into non-pharmacological, pharmacological, and standard and long-term agents, we will explore these aspects and highlight new evidence from the last 3 years.

## Standard therapeutic strategies

### Vaccination

There is an established role for the influenza and pneumococcal vaccinations in the management of chronic respiratory and medical conditions. This is based on limited and low-quality evidence ^12^. There have been no recent studies specific to bronchiectasis.

### Airway clearance techniques and pulmonary rehabilitation

Chest physiotherapy forms a core of patient-led management in bronchiectasis irrespective of severity and symptoms. These techniques are patient-centred and variable and aim to aid the removal of secretions from the lung through non-pharmacological methods ^13^. Whether these techniques have a clear impact on quality of life or reducing exacerbations has not been adequately proven, and most studies are limited to the CF patient population ^13^. Additionally, the use of multidisciplinary exercise interventions (pulmonary rehabilitation [PR]) is seen as an integral part of multimodal management for several chronic diseases ^14^. However, much of the data are based on CF or other chronic respiratory conditions. Further randomised controlled trials (RCTs) are required for PR specific to NCFB. Table 1 summarises the most recent studies and their outcomes for both RCTs.

The recent Cochrane review in 2015 included a total of seven studies in children and adults and a total of 105 patients. Overall, they showed within this limited field that airway clearance techniques (ACTs) are safe in adults and improve certain quality of life measures, lung function, and symptoms ^15^. Further studies are required to assess the short- and long-term impact on exacerbations and disease progression.

**Table 1.** Summary of randomised controlled trials (RCTs) for airway clearance techniques (ACTs).

Non-pharmacological therapy	Study	Study design	Results/outcome Lung function	Comments/ adverse events (AEs)
			Quality of life	
			Exercise capacity	
			Other	
**ACTs and PR**	Lee *et al.* ^38^	RCT PR and ACT versus control 43 PR and ACT monitoring 42 unsupervised exercise training but no ACT monitoring 8 weeks	No change in FEV1/FVC at study end	No data
			Secondary outcome: improved scores – LCQ	
			Primary endpoint: Short-term improvement in exercise capacity (shuttle walk – mean difference to control 62 m, 95% CI 24 to 101 m)	
			No significant difference between exacerbation rates/duration	
**ACTs**	Nicolini *et al.* ^39^ HFCWO versus chest physiotherapy (CPT) versus medical therapy only	RCT 10 subjects in each group (treatments up to 45 minutes for 5 days per week) 15 days	Secondary outcome: improved lung function ( *p* <0.001 and *p* <0.006)	No AEs
			Primary endpoint: symptom questionnaires Improvement of 2.7 in BCSS in HFCWO group ( *p* <0.001)	

			Increased sputum volume in the HFCWO and CPT group compared with control	

BCSS, breathlessness cough and sputum scale; CI, confidence interval; FEV _1_, forced expiratory volume in 1 second; FVC, forced vital capacity; HFCWO, high-frequency chest wall oscillation; LCQ, Leicester Cough questionnaire; PR, pulmonary rehabilitation.

## Short-term therapy

### Antimicrobial therapy

Fourteen days of antibiotic therapy is recommended for an acute exacerbation of bronchiectasis. The BTS guidelines identify an exacerbation as deterioration in local symptoms (cough, increased sputum volume or change of viscosity, increased sputum purulence with or without increasing wheeze, dyspnoea, and haemoptysis) and or systemic upset ^3^.

Intravenous (IV) antibiotics are required when there has been a failure of oral therapy or there is need for hospital admission or *in vitro* resistant pathogens that necessitate IV treatment. There were no studies addressing this. More evidence-based practice for choice and duration of antimicrobial therapy is required. The optimal management of exacerbations remains a vast area of untapped research for robust RCTs and future focus.

### Eradication therapy

Patients with known *Pseudomonas aeruginosa* (PA) have a reported severe phenotype with increased rates of exacerbation and an independent 3-fold mortality risk ^16^. This may be a result of the pathogenicity of the organism and its ability to form biofilms, rendering standard antimicrobial therapies less effective. Therefore, attempted eradication for first isolation is considered reasonable ^3^. Further studies are needed to assess 1) if eradication is needed and 2) the optimal management regime.

## Long-term therapies

### Muco-active therapies

Muco-active therapies can be used for both exacerbations and chronic management. Available as oral, inhaled, or nebulised agents, they reduce sputum viscosity and aid expectoration, thereby theoretically shortening exacerbation length or frequency and improving symptoms ^17^.

Although CF and previous studies have shown the potential benefit of hypertonic over 0.9% saline, more recently the evidence indicates equal efficacy in NCFB ^18^. Further studies are required to show whether saline (0.9% or hypertonic) is recommended in practice. Bilton *et al*. studied the effects of inhaled mannitol in a 12-month double-blinded RCT ^19^. There was no statistically significant reduction in exacerbation rates; however, there was an improvement in time to first exacerbation and quality of life indicators ^19^. The results are summarised in Table 2. Oral agents such as carbocisteine are commonly prescribed in the UK as part of bronchiectasis therapy; however, there are no RCTs to date. Dornase alfa therapy shows an increase in exacerbation rate in NCFB and is not recommended ^20^.

### Anti-inflammatory agents

This broad heading covers many drugs, including corticosteroids, non-steroidal anti-inflammatory drugs (NSAIDs), leukotriene receptor antagonists, and others. All have different mechanisms of action and vary as either long- or short-term therapy.

***NSAIDs.*** There is some evidence to suggest the use of ibuprofen or other NSAIDs in patients with mild CF ^21^. There are no studies that support their routine use in non-CF bronchiectasis.

***Leukotriene receptor antagonists.*** There are no studies to date for this subset of therapy specific to bronchiectasis.

***Inhaled corticosteroids +/- long acting beta 2 agonists.*** The Cochrane review of the long-acting beta _2_ agonists (LABA) and inhaled corticosteroids (ICS) combination demonstrated a lack of quality evidence ^22^. Only one RCT (2012) was highlighted in the 2014 review evaluating a bronchiectasis adult population without asthma who received inhaled budesonide and formoterol (640µg and 18µg) or high-dose budesonide (1,600µg). The authors found that there was an improvement in dyspnoea symptoms between the combination group and the ICS group ^23^. Study size was small and it lacked statistically significant differences in outcomes ^23^.

Predominant complications with long-term inhaled therapy are possible pneumonia risk, adrenal suppression, thin skin, and haemoptysis. This risk-benefit profile needs further investigation before management is accepted as routine in the bronchiectasis patient.

### Bronchodilator therapy

***Beta 2 adrenoreceptor agonists (short- and long-acting beta agonists).*** These agents have been illustrated in clinical trials for asthma and COPD; however, there have been none to date in bronchiectasis. Furthermore, there are no RCTs evaluating the use of inhaled anticholinergics. The role of bronchodilator therapy in bronchiectasis is unproven but often used in clinical practice with the breathless patient. If there is subjective improvement, it is sensible to continue such treatment.

### Other long-term therapies

Mandal *et al.* reported the use of atorvastatin versus placebo with the primary endpoint of reducing cough ^24^. Over a 6-month study period, the use of atorvastatin was shown to improve cough on quality of life questionnaire ^24^; the results are discussed in Table 2.

**Table 2.** Randomised controlled trial (RCT) on anti-inflammatory and muco-active agents.

Pharmacological therapy	Study and authors	Study design and intervention	Results Lung function Quality of life Exacerbations Others	Adverse events (AEs) or comments
**Anti-inflammatory** **agents**	Mandal *et al.* ^24^ Atorvastatin versus placebo	RCT Placebo-controlled 30 subjects atorvastatin 80 mg OD 30 subjects placebo OD 6 months	Spirometry: no change	Treatment group reported more headaches and gastrointestinal AEs
			Primary endpoint: improvement in cough – LCQ (mean difference 2.2, 95% CI 0.5–3.9; *p*=0.01)	
			Trend to reduced number of exacerbations	
			Reduced CRP Trend to improved incremental walk test	
**Muco-active** **agents**	Bilton *et al.* ^19^ Inhaled mannitol	RCT Placebo-controlled 233 subjects mannitol 400 mg BD 228 control low-dose mannitol 12 months	No endpoint data on lung function	AEs similar between groups
			Secondary endpoint: quality of life SGRQ improved in treatment arm	
			Primary endpoint: annual exacerbation rates No significant reduction 1.69 (95% CI 1.48–1.94) and 1.84 (95% CI 1.61–2.10), *p*=0.32.	
			Secondary endpoint: time to first exacerbation – improved in mannitol group (165 days versus 124 days [ *p*=0.022]) Duration of exacerbations: no change	

6MWT, 6-minute walk test; BD, *bis in die* (twice daily); CI, confidence interval; CRP, C-reactive protein; LCQ, Leicester Cough questionnaire; OD, *omni die* (every day); SGRQ, St George’s respiratory questionnaire.

Neutrophil elastase (NE) is a protease involved in inflammatory processes that has the ability to cause lung damage. It shows an increased activity in bronchiectasis. AZD9668 is an orally available NE inhibitor studied in a randomised, double-blinded, placebo-controlled phase II study by Stockley *et al*. ^25^. A total of 16 patients were randomised to placebo and 22 to treatment for 28 days ^25^. There was no statistical significance in sputum neutrophil count or weight. Secondary endpoint of quality of life questionnaire showed a clinical difference suggestive of benefit, but the results did not meet significance ^25^. Further studies on similar novel agents are encouraged in order to broaden our scope for long-term anti-inflammatory treatments.

### Macrolides

The use of chronic macrolide therapy has been described for many respiratory conditions. The mechanism of action has been the point of research topics and includes anti-inflammatory, immunomodulatory, and antimicrobial actions. Their chronic use has been noted within the diffuse panbronchiolitis and CF populations ^26^. This has spurred clinical trials in the bronchiectasis group.

Three major studies in 2012 and 2013 from Australia, New Zealand, and the Netherlands compared azithromycin daily, azithromycin three times weekly, and low-dose erythromycin and have led the way in establishing macrolide use in long-term bronchiectasis therapy ^27– 29^. Despite the publication date, all three studies (BLESS, BAT, and EMBRACE) have been included in this report because of their significance in current bronchiectasis management. The three trials reported their primary outcome as exacerbation frequency, and they have illustrated significant reduction in exacerbation rates ^27– 29^. There was some benefit in quality of life measures but a clinically insignificant improvement in lung function; Table 3 summarises the results.

**Table 3.** Summary of randomised controlled trials (RCTs) for long-term oral, inhaled, and nebulised antimicrobial therapies.

Pharmacological therapy	Study and authors	Study design and intervention	Results Lung function Quality of life Exacerbations	Adverse events (AEs) or comments
**Long-term** **macrolide therapy**	BAT (bronchiectasis and long-term azithromycin treatment) Altenburgh *et al.* ^27^ Azithromycin versus placebo	RCT Double-blinded Placebo-controlled 43 azithromycin 250 mg OD 40 matching placebo 12 months	Secondary endpoint: FEV _1_ 3-monthly Increased by 1.03% in treatment group and decreased by 0.10% in placebo group ( *p*=0.47)	GI AEs: 40% in treatment group and 5% in placebo group Macrolide resistance to oropharyngeal flora: 88% in treatment group and 26% in placebo group oropharyngeal flora
			Secondary endpoint: SGRQ Improved by 6 units per 6 months in treatment group and 2 units in placebo group ( *p*=0.046)	
			Primary endpoint: median number of exacerbations in 12 months Azithromycin group 0, placebo group 2 ( *p* <0.001)	
	BLESS (bronchiectasis and low-dose erythromycin study) Serisier *et al.* ^28^ Erythromycin versus placebo	RCT Double-blinded Placebo-controlled 59 subjects 400 mg BD erythromycin 58 subjects matching placebo 12 months	Secondary outcome: reduction in rate of decline of FEV _1_ No statistically significant difference	Statistically significant increase in macrolide resistance (oropharyngeal flora) in treatment vs. placebo group (secondary endpoint)
			Secondary endpoint: SGRQ –1.3 units in placebo group and –3.9 units in treatment group; no statistically significant difference	
			Primary endpoint: annualised mean rate of exacerbations Statistically significant reduction: mean 1.29 [95% CI 0.93–1.65] versus 1.97 [95% CI 1.45–2.48] erythromycin versus placebo	
	EMBRACE (azithromycin for the prevention of exacerbations) Wong *et al.* ^29^ Azithromycin versus placebo	RCT Double-blinded Placebo-controlled 71 subjects azithromycin 500 mg M/W/F 70 subjects placebo equivalent 6 months	Co-primary endpoint: FEV _1_ before bronchodilator There was no statistically significant difference	More frequent GI side effects in treatment group No routine macrolide resistance testing
			Co-primary endpoint: SGRQ No statistically significant difference between groups at 12-month follow up	
			Primary endpoint: exacerbation rate 42 event-based exacerbations in treatment group versus 102 in placebo group. Rate was 0.59 in treatment group versus 1.57 in placebo group (rate ratio 0.38, 95% CI 0.26–0.54; *p*<0.0001).	
**Inhaled and** **nebulised** **antimicrobial therapy**	ORBIT II (once-daily respiratory bronchiectasis inhalation treatment) Serisier *et al.* ^35^ Inhaled ciprofloxacin versus placebo	RCT Double-blinded Placebo-controlled 20 subjects dual release ciprofloxacin for inhalation via nebuliser 3 cycles versus placebo 22 OD 3 cycles 28 days	No difference in FEV _1_	No difference in AEs
			No difference in quality of life (SGRQ) Secondary endpoint: increased time to first exacerbation Median 134 versus 58 days, p=0.057 (nITT) but 0.046 (per protocol)	
			Primary endpoint: Mean (SD) 4.2 (3.7) log _10_ CFU/g reduction in PA bacterial density at day 28 (versus −0.08 [3.8] with placebo) *p*=0.002	
	ORBIT III and IV (NCT01515007 and NCT02104245) O’Donnell *et al.* ^37^ Dual-release inhaled pulmaquin	RCT Double-blinded Placebo-controlled 584 subjects randomised 6 cycles of 28 days on and off over 48 weeks		
			Secondary endpoint: quality of life	
			Primary endpoint: time to first exacerbation – results to be published Secondary endpoints: number of exacerbations/severe exacerbations	
	RESPIRE I De Soyza *et al.* ^34^ Inhaled ciprofloxacin	RCT Double-blinded Placebo-controlled Ciprofloxacin DPI 32.5 mg versus placebo 416 patients randomised 2 regimens: 14 days on/off or 28 days on/off for 48 weeks		No significant difference in adverse drug reactions between both groups reported in abstract

			Primary endpoint: time to first exacerbation Significant prolongation in treatment versus placebo group ( *p*=0.0005). Reduced frequency of exacerbations ( *p*=0.0061).	
	AIR-BX1 AIR-BX2 Barker *et al.* ^31^ Inhaled aztreonam versus placebo	RCT x 2 Double-blinded Placebo-controlled 1) 134 AZLI 75 mg TDS for 4 weeks with 4 week off periods, 132 placebo 2) 136 AZLI and 138 placebo 75 mg TDS for 4 weeks >with 4 week off periods 2 cycles		More AEs (increased cough, sputum, and dyspnoea) reported in treatment versus placebo
			Primary endpoint: reduction in bronchiectasis symptoms (QOL-B-RSS) No difference in AIR-BX1. Difference in AIR-BX2: 4.6 (1.1 to 8.2), *p*=0.011.	

	Wilson *et al.* ^40^ Inhaled ciprofloxacin DPI versus placebo	RCT Double-blinded Placebo-controlled 60 subjects ciprofloxacin DPI 32.5 mg BD 64 subjects BD placebo 28 days	Secondary endpoint: Treatment FEV _1_ improves by 0.06 ± 8.36% Placebo FEV _1_ decreased by –0.40 ± 10.36% No statistically significant difference	50 versus 54 in placebo of any reported AEs
			Secondary endpoints: improvement in SGRQ Adjusted mean difference between treatment and placebo was –3.56 (95% CI –7.3–0.1, *p*=0.059)	
			22 treatment subjects versus 25 placebo reported ≥1 exacerbation	
			Primary endpoint: reducing bacterial load Significant reduction in CFU (–3.62 log _10_ CFU/g, range –9.78–5.02 log _10_ CFU/g) versus placebo (–0.27 log _10_ CFU/g, range –7.96–5.25 log _10_ CFU/g; *p* <0.001)	
	RESPIRE 1 and 2 (NCT01764841) Inhaled ciprofloxacin versus placebo	RCT Double-blinded Placebo-controlled Ciprofloxacin inhaler BD 14 days and 28 days versus placebo (on and off period) 12 months	Results to be published	Results to be published
			Secondary endpoints: FEV _1_, quality of life (SGRQ), number of exacerbations, eradicated and new pathogens	
			Primary endpoint: time to first exacerbation	
	Haworth *et al.* ^32^ Nebulised colistin versus nebulised saline	RCT Double-blinded Placebo-controlled 73 colistin 1 million IU BD 71 placebo (0.45% saline) BD 6 months	FEV _1_ mean differences at 4, 12, and 26 weeks: –0.05, –0.11, and –0.10L Treatment differences: no statistical significance 95% CI –0.17 to 0.07, –0.17 to 0.07, and –0.22 to 0.02	Total 143 AEs in treatment versus 108 in placebo. Five colistin patients withdrew because of bronchoconstriction
			SGRQ at baseline, week 12, and week 26 Mean difference at 12 weeks’ colistin: –2.8 Units and placebo –2.2 Units Week 26 colistin: –10.4 and placebo –0.4 Reached statistical significance at week 26 ( *p*=0.006)	
			Primary endpoint: time to first exacerbation Median time 168 days with colistin versus 103 days with placebo ( *p*=0.038) in adherent patients Importance of adherence emphasised	
			Secondary endpoints: severity of exacerbations, adherence, sputum weight, and CFUs of PA Significant reduction in PA density in treatment group versus placebo group	

AZLI, aztreonam for inhalation solution; BD, *bis in die* (twice daily); CFU, colony-forming units; CI, confidence interval; DPI, dry powder for inhalation; FEV _1_, forced expiratory volume in 1 second; GI, gastrointestinal; IU, international unit; OD, *omni die* (every day); PA, *Pseudomonas aeruginosa*; QOL-B-RSS, quality of life-bronchiectasis respiratory symptoms score; SGRQ, St George’s respiratory questionnaire; TDS, *ter die sumendum* (three times a day).

Although long-term antibiotics are still not recommended on a routine basis, in a selected group based on frequent exacerbations (three or more per annum) or fewer exacerbations but increasing morbidity, this intervention should be considered. However, we must balance this with the possible emergence of antibiotic resistance and possible cardiovascular, audiologic, and gastrointestinal adverse events (AEs). A practical but prudent issue is the necessity to screen these patients for NTM infection prior to embarking upon long-term macrolide therapy, as this would have potential consequence on NTM management ^30^.

The prospect of non-antimicrobial therapies and novel anti-inflammatories exposes an exciting area for future phase II and III clinical trials.

### Inhaled or aerosolised antibiotic or other therapies

With the burden of antimicrobial resistance increasingly real and a global political agenda for stewardship, the need for enhancing techniques and the delivery of antimicrobials responsibly with reduced adverse effects is paramount. Aerosolised and inhaled delivery within the lung has been of interest, as high concentrations of drug can be administered within the airways with reduced systemic side effects. The basis for inhaled antibiotics in bronchiectasis has not yet been established in clinical trials; however, recent work is exposing an exciting field. Currently, there are no approved inhaled antibiotics licensed for use by UK, European, or USA drug agencies for bronchiectasis.

Barker *et al.* studied the effects of inhaled aztreonam in bronchiectasis patients in two double-blinded, randomised, placebo-controlled trials ^31^ ( Table 3). Their primary endpoint of quality of life did not reach statistical significance, and they also reported more treatment-related AEs ^31^.

Table 3 illustrates several RCTs analysing the safety and effect of inhaled or nebulised agents in long-term therapy. Haworth *et al.* studied the effectiveness of inhaled colistin for up to 6 months within 21 days of an exacerbation ^32^. Although they did show a difference in the median time to first exacerbation, it did not reach statistical significance. However, improvements in secondary endpoints of bacterial density and quality of life did. This study also illustrated the importance of adherence, and, when taking more than 80% of therapy, it did improve the time to first exacerbation significantly ^32^.

There are two phase III trials of inhaled ciprofloxacin, RESPIRE-1 and -2, that have now been completed with the results awaited. They have two regimens comparing 28 days on and off for 1 year versus 14 days on and off for 1 year. A competitor study has utilised liposomes to allow slow release of ciprofloxacin and molecular stability in nebulisation and to improve delivery to macrophages ^33, 34^. Successful phase II studies of liposomal ciprofloxacin (pulmaquin) have shown a significant reduction in PA colony-forming units (CFUs) in sputum compared with placebo ^35^. Subsequent to this, two identical international studies have completed enrolment to a phase III study (ORBIT-3 and -4) ^36, 37^. The primary endpoint will be evaluation of time to first exacerbation, and secondary endpoints include quality of life measures ^37^. Overall, the initial results are encouraging, prove tolerability, and promise a potential inhaled therapy for bronchiectasis ( Table 3). The full reported results of ongoing phase III studies in inhaled ciprofloxacin and nebulised ciprofloxacin with liposomal technology are eagerly awaited.

## Summary

There has been increased research activity in the field of bronchiectasis owing to its growing burden on healthcare systems secondary to reported increased prevalence. However, the quality of evidence in the field remains limited owing to the lack of RCTs. Long-term RCTs are greatly needed to further this field and improve patient outcome.

## References

[1] Chalmers JD Aliberti S Blasi F: Management of bronchiectasis in adults. *Eur Respir J.* 2015;45(5):1446–62. 10.1183/09031936.00119114 25792635

[2] Bilton D: Update on non-cystic fibrosis bronchiectasis. *Curr Opin Pulm Med.* 2008;14(6):595–9. 10.1097/MCP.0b013e328312ed8c 18812838

[3] Pasteur MC Bilton D Hill AT: British Thoracic Society guideline for non-CF bronchiectasis. *Thorax.* 2010;65(7):577. 10.1136/thx.2010.142778 20627912

[4] Quint JK Millett ER Joshi M: Changes in the incidence, prevalence and mortality of bronchiectasis in the UK from 2004 to 2013: a population-based cohort study. *Eur Respir J.* 2016;47(1):186–93. 10.1183/13993003.01033-2015 26541539 PMC4982534

[5] Lonni S Chalmers JD Goeminne PC: Etiology of Non-Cystic Fibrosis Bronchiectasis in Adults and Its Correlation to Disease Severity. *Ann Am Thorac Soc.* 2015;12(12):1764–70. 10.1513/AnnalsATS.201507-472OC 26431397 PMC5467084

[6] Hill A Bilton D Brown J: British thoracic society quality standards for clinically significant bronchiectasis in adults July 2012. *Br Thorac Soc Rep.* 2012;4:1–16. Reference Source

[7] Guan WJ Gao YH Xu G: Aetiology of bronchiectasis in Guangzhou, southern China. *Respirology.* 2015;20(5):739–48. 10.1111/resp.12528 25819403

[8] Loebinger MR Wells AU Hansell DM: Mortality in bronchiectasis: a long-term study assessing the factors influencing survival. *Eur Respir J.* 2009;34(4):843–9. 10.1183/09031936.00003709 19357155

[9] Chalmers JD Goeminne P Aliberti S: The bronchiectasis severity index. An international derivation and validation study. *Am J Respir Crit Care Med.* 2014;189(5):576–85. 10.1164/rccm.201309-1575OC 24328736 PMC3977711

[10] Martínez-García MÁ de Gracia J Vendrell Relat M: Multidimensional approach to non-cystic fibrosis bronchiectasis: the FACED score. *Eur Respir J.* 2014;43(5):1357–67. 10.1183/09031936.00026313 24232697

[11] Cole PJ: Inflammation: a two-edged sword--the model of bronchiectasis. *Eur J Respir Dis Suppl.* 1986;147:6–15. 3533593

[12] Chang CC Singleton RJ Morris PS: Pneumococcal vaccines for children and adults with bronchiectasis. *Cochrane Database Syst Rev.* 2007; (2): CD006316. 10.1002/14651858.CD006316.pub2 17443619

[13] McCool FD Rosen MJ: Nonpharmacologic airway clearance therapies: ACCP evidence-based clinical practice guidelines. *Chest.* 2006;129(1 Suppl):250S–9. 10.1378/chest.129.1_suppl.250S 16428718

[14] Burtin C Hebestreit H: Rehabilitation in patients with chronic respiratory disease other than chronic obstructive pulmonary disease: exercise and physical activity interventions in cystic fibrosis and non-cystic fibrosis bronchiectasis. *Respiration.* 2015;89(3):181–9. 10.1159/000375170 25676797

[15] Lee AL Burge AT Holland AE: Airway clearance techniques for bronchiectasis. *Cochrane Database Syst Rev.* 2015; (11): CD008351. 10.1002/14651858.CD008351.pub3 26591003 PMC7175838

[16] Finch S McDonnell MJ Abo-Leyah H: A Comprehensive Analysis of the Impact of *Pseudomonas aeruginosa* Colonization on Prognosis in Adult Bronchiectasis. *Ann Am Thorac Soc.* 2015;12(11):1602–11. 10.1513/AnnalsATS.201506-333OC 26356317

[17] Wilkinson M Sugumar K Milan SJ: Mucolytics for bronchiectasis. *Cochrane Database Syst Rev.* 2014; (5):CD001289. 10.1002/14651858.CD001289.pub2 24789119 PMC6513404

[18] Nicolson CH Stirling RG Borg BM: The long term effect of inhaled hypertonic saline 6% in non-cystic fibrosis bronchiectasis. *Respir Med.* 2012;106(5):661–7. 10.1016/j.rmed.2011.12.021 22349069

[19] Bilton D Tino G Barker AF: Inhaled mannitol for non-cystic fibrosis bronchiectasis: a randomised, controlled trial. *Thorax.* 2014;69(12):1073–9. 10.1136/thoraxjnl-2014-205587 25246664

[20] O'Donnell AE Barker AF Ilowite JS: Treatment of idiopathic bronchiectasis with aerosolized recombinant human DNase I. rhDNase Study Group. *Chest.* 1998;113(5):1329–34. 10.1378/chest.113.5.1329 9596315

[21] Pizzutto SJ Upham JW Yerkovich ST: Inhaled non-steroid anti-inflammatories for children and adults with bronchiectasis. *Cochrane Database Syst Rev.* 2016; (1):CD007525. 10.1002/14651858.CD007525.pub3 26816298 PMC9444006

[22] Goyal V Chang AB: Combination inhaled corticosteroids and long-acting beta _2_-agonists for children and adults with bronchiectasis. *Cochrane Database Syst Rev.* 2014; (6):CD010327. 10.1002/14651858.CD010327.pub2 24913725 PMC6483496

[23] Martínez-García MÁ Soler-Cataluña JJ Catalán-Serra P: Clinical efficacy and safety of budesonide-formoterol in non-cystic fibrosis bronchiectasis. *Chest.* 2012;141(2):461–8. 10.1378/chest.11-0180 21778259

[24] Mandal P Chalmers JD Graham C: Atorvastatin as a stable treatment in bronchiectasis: a randomised controlled trial. *Lancet Respir Med.* 2014;2(6):455–63. 10.1016/S2213-2600(14)70050-5 24717640

[25] Stockley R De Soyza A Gunawardena K: Phase II study of a neutrophil elastase inhibitor (AZD9668) in patients with bronchiectasis. *Respir Med.* 2013;107(4):524–33. 10.1016/j.rmed.2012.12.009 23433769

[26] Gao YH Guan WJ Xu G: Macrolide therapy in adults and children with non-cystic fibrosis bronchiectasis: a systematic review and meta-analysis. *PLoS One.* 2014;9(3):e90047. 10.1371/journal.pone.0090047 24603554 PMC3946068

[27] Altenburg J de Graaff CS Stienstra Y: Effect of azithromycin maintenance treatment on infectious exacerbations among patients with non-cystic fibrosis bronchiectasis: the BAT randomized controlled trial. *JAMA.* 2013;309(12):1251–9. 10.1001/jama.2013.1937 23532241

[28] Serisier DJ Martin ML McGuckin MA: Effect of long-term, low-dose erythromycin on pulmonary exacerbations among patients with non-cystic fibrosis bronchiectasis: the BLESS randomized controlled trial. *JAMA.* 2013;309(12):1260–7. 10.1001/jama.2013.2290 23532242

[29] Wong C Jayaram L Karalus N: Azithromycin for prevention of exacerbations in non-cystic fibrosis bronchiectasis (EMBRACE): a randomised, double-blind, placebo-controlled trial. *Lancet.* 2012;380(9842):660–7. 10.1016/S0140-6736(12)60953-2 22901887

[30] Spreading US Macrolide Resistance. *JAMA.* 2015;314(12):1218 10.1001/jama.2015.11206

[31] Barker AF O'Donnell AE Flume P: Aztreonam for inhalation solution in patients with non-cystic fibrosis bronchiectasis (AIR-BX1 and AIR-BX2): two randomised double-blind, placebo-controlled phase 3 trials. *Lancet Respir Med.* 2014;2(9):738–49. 10.1016/S2213-2600(14)70165-1 25154045

[32] Haworth CS Foweraker JE Wilkinson P: Inhaled colistin in patients with bronchiectasis and chronic *Pseudomonas aeruginosa* infection. *Am J Respir Crit Care Med.* 2014;189(8):975–82. 10.1164/rccm.201312-2208OC 24625200 PMC4098097

[33] Cipolla D Blanchard J Gonda I: Development of Liposomal Ciprofloxacin to Treat Lung Infections. *Pharmaceutics.* 2016;8(1): pii: E6. 10.3390/pharmaceutics8010006 26938551 PMC4810082

[34] De Soyza A Aksamit T Bandel TJ: LATE-BREAKING ABSTRACT: RESPIRE 1: Ciprofloxacin DPI 32.5 mg bd administered 14 day on/off or 28 day on/off *vs*. placebo for 48 weeks in subjects with non-cystic fibrosis bronchiectasis (NCFB). *Eur Respir J.* 2016 48(suppl 60):OA272 10.1183/13993003.congress-2016.OA272

[35] Serisier DJ Bilton D De Soyza A: Inhaled, dual release liposomal ciprofloxacin in non-cystic fibrosis bronchiectasis (ORBIT-2): a randomised, double-blind, placebo-controlled trial. *Thorax.* 2013;68(9):812–7. 10.1136/thoraxjnl-2013-203207 23681906 PMC4770250

[36] Bilton D Serisier D Wanner A: Orbit-3 And Orbit-4: Design Of A Phase 3 Program To Investigate Safety And Efficacy Of Pulmaquin® In Non-Cystic Fibrosis Bronchiectasis (ncfbe) Patients Chronically Colonized With *Pseudomonas Aeruginosa* (pa). *Am J Respir Crit Care Med.* 193:p.2016.

[37] O'Donnell AE Bilton D Serisier D: ORBIT-3 And ORBIT-4: Design Of A Phase 3 Program To Investigate Safety And Efficacy Of Pulmaquin® In Non-Cystic Fibrosis Bronchiectasis (NCFBE) Patients Chronically Colonized With *Pseudomonas Aeruginosa* (PA). In A51. BRONCHIECTASIS: CLINICAL AND EPIDEMIOLOGIC STUDIES. American Thoracic Society.2016;193:A1775–A1775. Reference Source

[38] Lee AL Hill CJ Cecins N: The short and long term effects of exercise training in non-cystic fibrosis bronchiectasis--a randomised controlled trial. *Respir Res.* 2014;15(1):44. 10.1186/1465-9921-15-44 24731015 PMC3996132

[39] Nicolini A Cardini F Landucci N: Effectiveness of treatment with high-frequency chest wall oscillation in patients with bronchiectasis. *BMC Pulm Med.* 2013;13:21. 10.1186/1471-2466-13-21 23556995 PMC3623823

[40] Wilson R Welte T Polverino E: Ciprofloxacin dry powder for inhalation in non-cystic fibrosis bronchiectasis: a phase II randomised study. *Eur Respir J.* 2013;41(5):1107–15. 10.1183/09031936.00071312 23018904 PMC3640146

